# Quantitative Mass Spectrometry Analysis Reveals Similar Substrate Consensus Motif for Human Mps1 Kinase and Plk1

**DOI:** 10.1371/journal.pone.0018793

**Published:** 2011-04-13

**Authors:** Zhen Dou, Conrad von Schubert, Roman Körner, Anna Santamaria, Sabine Elowe, Erich A. Nigg

**Affiliations:** 1 Department of Cell Biology, Max Planck Institute of Biochemistry, Martinsried, Germany; 2 Biozentrum, University of Basel, Basel, Switzerland; 3 Hefei National Laboratory of Physical Sciences at the Microscale, University of Science and Technology of China, Hefei, China; University of Texas-Houston Medical School, United States of America

## Abstract

**Background:**

Members of the Mps1 kinase family play an essential and evolutionarily conserved role in the spindle assembly checkpoint (SAC), a surveillance mechanism that ensures accurate chromosome segregation during mitosis. Human Mps1 (hMps1) is highly phosphorylated during mitosis and many phosphorylation sites have been identified. However, the upstream kinases responsible for these phosphorylations are not presently known.

**Methodology/Principal Findings:**

Here, we identify 29 *in vivo* phosphorylation sites in hMps1. While *in vivo* analyses indicate that Aurora B and hMps1 activity are required for mitotic hyper-phosphorylation of hMps1, *in vitro* kinase assays show that Cdk1, MAPK, Plk1 and hMps1 itself can directly phosphorylate hMps1. Although Aurora B poorly phosphorylates hMps1 *in vitro*, it positively regulates the localization of Mps1 to kinetochores *in vivo*. Most importantly, quantitative mass spectrometry analysis demonstrates that at least 12 sites within hMps1 can be attributed to autophosphorylation. Remarkably, these hMps1 autophosphorylation sites closely resemble the consensus motif of Plk1, demonstrating that these two mitotic kinases share a similar substrate consensus.

**Conclusions/Significance:**

hMps1 kinase is regulated by Aurora B kinase and its autophosphorylation. Analysis on hMps1 autophosphorylation sites demonstrates that hMps1 has a substrate preference similar to Plk1 kinase.

## Introduction

The purpose of mitosis is to equally distribute the duplicated genome amongst dividing cells. Defects in chromosome segregation can lead to aneuploidy, which in turn is implicated in tumorgenesis [1], [2]. Attachment of mitotic chromosomes to spindle microtubules is mediated by the kinetochore (KT), a proteinaceous complex assembled on centromeres [3], [4]. Importantly, the KT functions not only as a structural platform, but also as a signaling hub to coordinate chromosome attachment, SAC activity and the metaphase to anaphase transition [5]. The purpose of the SAC signaling cascade is to delay the onset of anaphase until all chromosomes have undergone stable, bi-oriented attachments [6], [7]. Biochemically, this is achieved through inhibition of the ubiquitin ligase APC/C (anaphase promoting complex/cyclosome), in part through sequestration of Cdc20, an activator protein of the APC/C [7], [8].

SAC function requires several proteins that localize to the outer KT. Of these, Bub1, Bub3, Mad1, Mad2, Mad3/BubR1 and Mps1 are conserved from yeast to humans [6]. Mps1 (‘monopolar spindle 1’) was originally identified in budding yeast as a gene required for spindle pole body (SPB) duplication [9]. Subsequently, Mps1 was found also to be essential for SAC activity [10], [11] and this latter function is clearly conserved in evolution [12], [13], [14], [15], [16]. Human Mps1 (hMps1; also known as TTK [17]) peaks in expression and activity as cells go through mitosis [14], [18]. Moreover, mammalian cells depleted of Mps1 are unable to sustain full SAC activity [14], [19]. hMps1 is known to recruit checkpoint components to unattached kinetochores, including Mad1 and Mad2 [13], [16], [20], [21], [22], [23], [24], and to stabilize APC/C inhibitory complexes [22]. Furthermore, hMps1 was found to contribute to the correction of improper chromosome attachments [20], [22], [23], [25], [26], echoing earlier studies in budding yeast [27], [28]. When the SAC is active during prometaphase, hMps1 is hyper-phosphorylated, concomitant with high hMps1 activity [14]. It has been demonstrated that hMps1 autophosphorylates on T676 within the activation loop and that this modification is required for full activity *in vitro* [29] and SAC function *in vivo* [30], [31]. Most recently, *in vitro* autophosphorylation of the catalytic domain of hMps1 on at least 16 residues was described and phosphorylations at both T676 and T686 were shown to be important for catalytic activity [32]. In addition, several *in vivo* phosphorylation sites outside the catalytic domain have been described [30], [31], [33], but the functional significance of these latter phosphorylations has not yet been studied in detail.

Here, we have explored which upstream kinases might be responsible for phosphorylation of hMps1 during mitosis. We identified 29 *in vivo* phosphorylation sites within hMps1, including 4 sites that have not previously been reported. While Cdk1, MAPK, Plk1 and hMps1 itself can phosphorylate hMps1 *in vitro*, the kinase activities of Aurora B and hMps1 are required for the hyper-phosphorylation induced upshift (retardation of the gel electrophoretic mobility) of hMps1 during mitosis. Using quantitative mass spectrometry, we demonstrate that numerous phosphorylation sites, including several sites matching the Plk1 consensus, are in fact hMps1 autophosphorylation sites. This indicates that hMps1 and Plk1 are able to phosphorylate similar motifs.

## Results

### Identification of *in vivo* phosphorylation sites on hMps1 kinase

**Figure 1:**
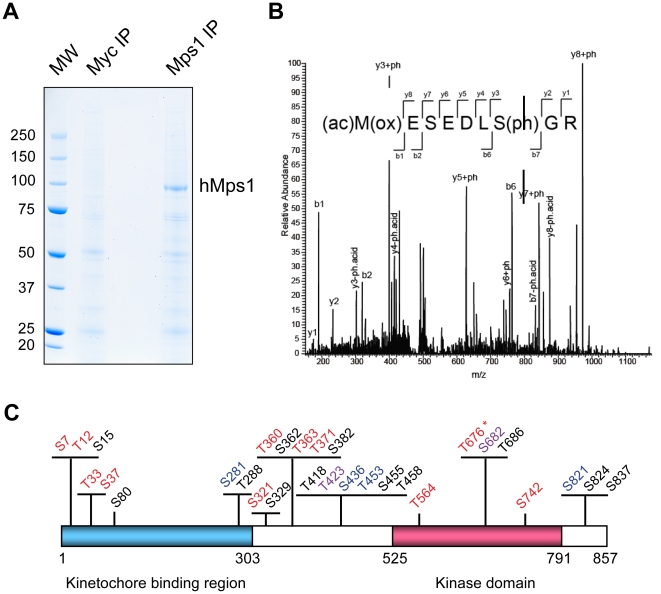
Identification of *in vivo* hMps1 phosphorylation sites. (A) Coomassie Brilliant Blue (CBB) staining of anti-Myc and anti-hMps1 immunoprecipitation products resolved on a 4–12% NuPAGE gel. About 20 mg mitotic HeLa S3 cell lysate was incubated with anti-Myc or anti-hMps1 N1 antibody coupled with protein G beads, respectively. (B) Collision induced dissociation (CID) mass spectrum of the human TTK/hMps1 derived phosphopeptide (1–9) (ac)M(ox)ESEDLS(ph)GR. C-terminal and N-terminal fragments of the peptide are marked as y- and b-ions, respectively, S(ph) denotes phospho-serine, and (ac)M(ox) represents the acetylated and oxidized N-terminus. The observed peptide fragments are also shown within the sequence above the spectrum. The MS/MS spectrum unambiguously identifies S7 as the phosphorylated amino acid within the peptide. (C) Schematic showing hMps1 phosphorylation sites identified by mass spectrometry. Sites conforming or resembling to a Plk1, Cdk1/MAPK and Aurora B consensus motifs are shown in red, blue and purple, respectively. The asterisk indicates the phosphorylation site in the activation loop.

To study the functional significance of hMps1 phosphorylation, we first carried out experiments aimed at mapping phosphorylation sites on hMps1 during mitosis. Using a previously described anti-hMps1-N1 monoclonal antibody (mAb) [14], we immunoprecipitated endogenous hMps1 protein from Nocodazole arrested mitotic HeLa S3 cell lysates. A band of 97 kDa, the predicted molecular weight of hMps1 (Fig. 1A), was subjected to in-gel digestion with trypsin, TiO_2_ based enrichment of phosphopeptides and analysis by mass spectrometry. A total of 29 phosphorylation sites were identified (Fig. 1B for a representative spectrum). Of these, 4 sites (S329, T418, T423 or T424, T458) represent novel sites. Moreover, 3 sites (T564, S682, S742) previously reported to be phosphorylated *in vitro* are shown here to be phosphorylated also *in vivo*. The remaining 22 sites were independently identified by other groups during the course of our study (Table S1) [30], [31], [33].

As a first step towards understanding which kinase(s) might be responsible for the observed phosphorylations, the 29 phosphorylation sites identified in this study were grouped according to known consensus motifs for key mitotic kinases. Four sites (S281, S436, T453, S821) matched an S/T-P motif, suggesting that they are likely targets of proline-directed kinases, notably CDKs or MAPKs [34]. Three sites (T12, S37, S363) conform to the classic Plk1 kinase consensus motif D/E-X-S/T-φ [35], and two additional sites (S321 and T564) contain a N/Q in position −2 as well as a hydrophobic residue in position +1. These latter sites conform to a broadened Plk1 consensus motif [36]. In addition, six other sites (S7, T33, T360, T371, T676, S742) share features with the broadened Plk1 consensus motif (E/D/N/Q at −2 position), although they lack a hydrophobic residue at position +1. Finally, S682 qualifies as a potential Aurora B phosphorylation site [37], but the remaining 13 sites do not match the consensus for any of the above kinases (Table S1). The positioning of phosphorylation sites along the primary sequence of hMps1 is illustrated in Fig. 1C.

### hMps1 is a likely substrate of several mitotic kinases

**Figure 2:**
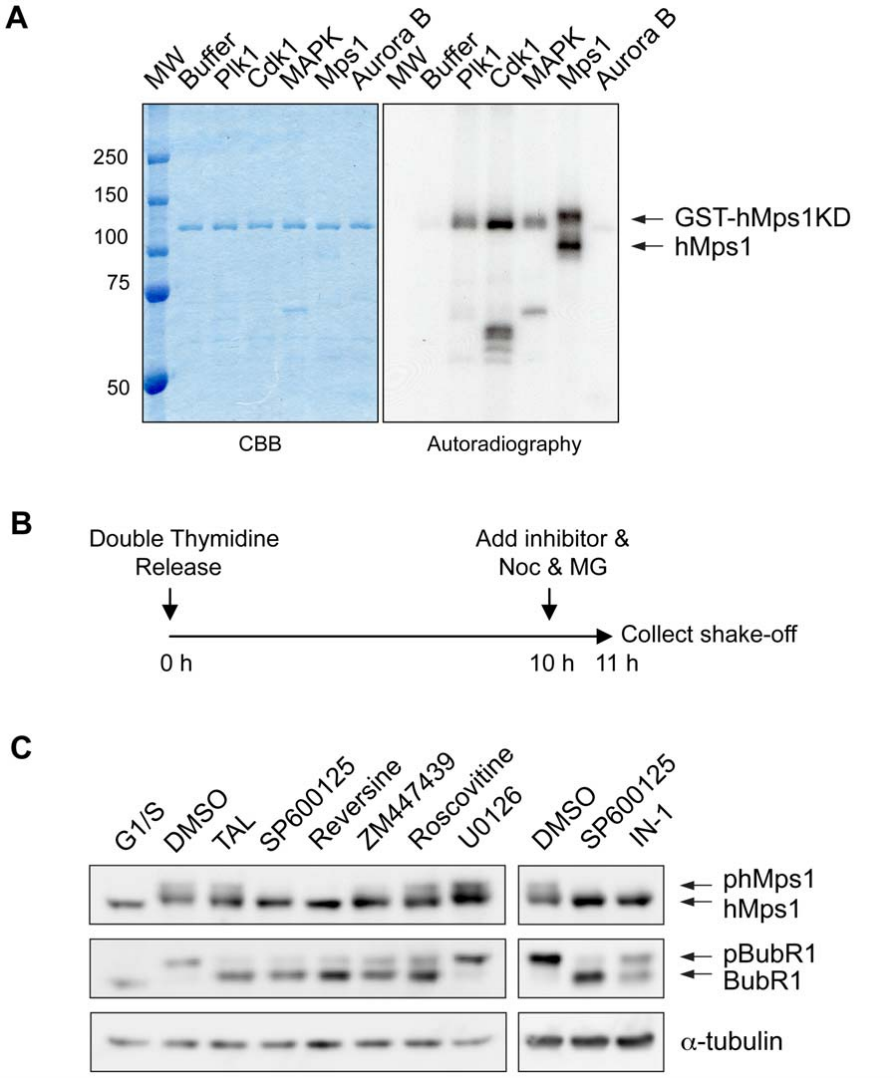
hMps1 is a likely substrate of several mitotic kinases. (A) *In vitro* phosphorylation of recombinant GST-hMps1KD in either kinase buffer alone, or by recombinant Plk1, Cdk1, MAPK, Aurora B kinase and immuno-purified hMps1 kinase. The left panel shows CBB staining of the SDS-PAGE gel, the right panel shows the result of autoradiography. (B) Schematic representation of the protocol followed to analyze the upstream kinase(s) required for hMps1 hyperphosphorylation *in vivo*, as monitored through a characteristic upshift. (C) Mitotic cells treated with the indicated small molecule inhibitors were collected using the protocol shown in (B) and processed for Western blotting against hMps1, BubR1 and α-tubulin respectively.

The observation that phosphorylation sites identified on endogenous hMps1 conform to the consensus motifs of several known mitotic kinases raised the possibility that hMps1 is regulated by one or more of these enzymes. To directly address this question, a kinase dead (KD) version of recombinant full-length GST-hMps1 protein was purified from Sf9 cells and used as an *in vitro* substrate for a panel of recombinant kinases. As shown in Fig. 2A, GST-hMps1-KD could readily be phosphorylated by Cdk1/Cyclin B as well as, albeit to a lesser extent, both MAPK (Erk2) and Plk1. In addition, immuno-purified hMps1 kinase also phosphorylated GST-hMps1-KD, as well as itself (Fig. 2A). In contrast, Aurora B barely phosphorylated GST-hMps1-KD, although it strongly phosphorylated MCAK (Fig. S1), a known Aurora B substrate [38], [39].

To determine whether any of the above kinases also phosphorylates hMps1 *in vivo*, we used Western blotting to monitor the effects of various kinase inhibitors on the phosphorylation-induced mitotic upshift of endogenous hMps1. As illustrated in Fig. 2B, cells were released from a double Thymidine arrest and 10 hours later treated for 1 hour with either DMSO or kinase inhibitors, together with Nocodazole and the proteasome inhibitor MG132 (to ensure a mitotic arrest). When compared to hMps1 in Thymidine arrested cells, the migration of hMps1 in DMSO-treated mitotic cells was clearly retarded, and, as shown previously, this upshift reflects hMps1 hyperphosphorylation [14]. The upshift was also observed after inhibition of Plk1 by TAL [40], Cdk1 by Roscovitine, or MAPK by U0126 (Fig. 2C). In contrast, the upshift was significantly attenuated in the presence of the hMps1 inhibitors SP600125 [41], Reversine [21] and Mps1-IN-1 [16] or the Aurora B inhibitor ZM447439 [42]. To demonstrate the efficacy of drug treatment, a mitotic upshift of BubR1 was monitored in parallel. In agreement with previous results [43], [44], the BubR1 upshift was attenuated in response to TAL, Roscovitine, or ZM447439; interestingly, it was sensitive also to SP600125, Reversine and Mps1-IN-1. Taken together, these results suggest that hMps1 itself as well as Aurora B control the hyperphosphorylation-induced upshift that is typical of hMps1 during mitosis. Considering that hMps1 was a poor *in vitro* substrate of Aurora B (Fig. 2A), Aurora B inhibition is likely to reduce the upshift of hMps1 through an indirect mechanism.

**Figure 3:**
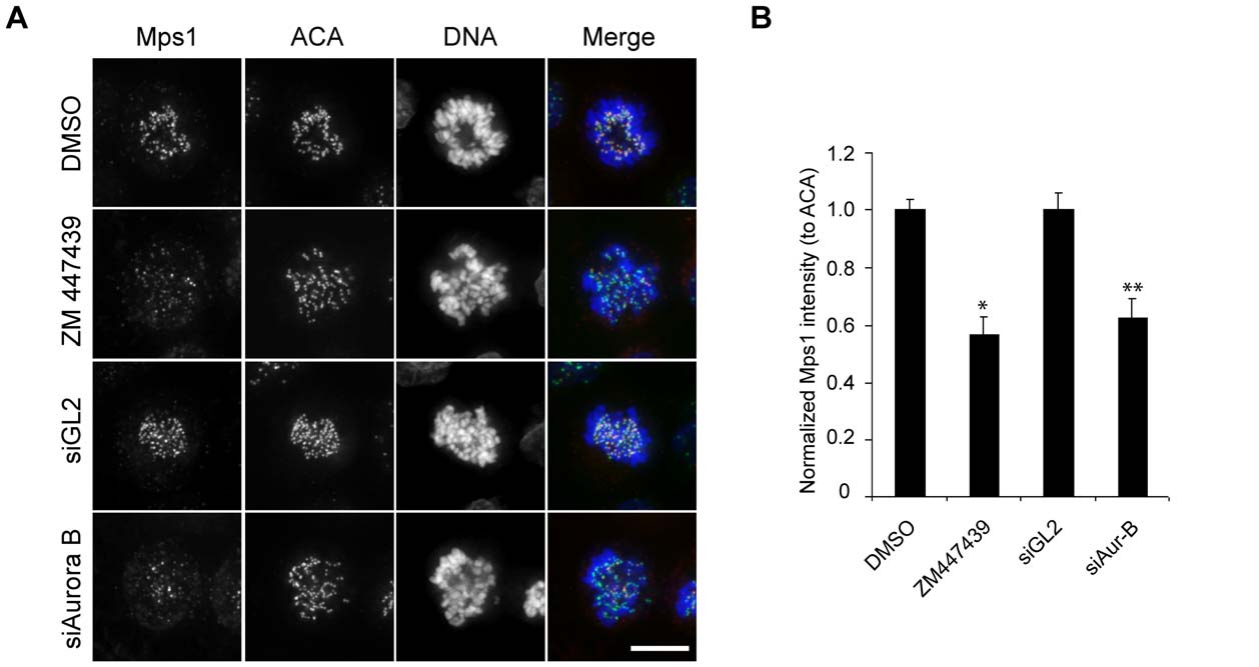
Aurora B is required for maximal KT localization of hMps1. (A) Representative immunofluorescence images of prometaphase cells treated for 1 hour with DMSO or ZM447439, or transfected with GL2- or Aurora-B-directed siRNA. Thirty-six hours after transfection, cells were fixed with PHEM buffer and then co-stained for Mps1 (red), ACA (green), and DNA (blue). Scale bar represents 10 µm. (B) Bar graph showing the quantification of hMps1 KT signal (normalized to ACA signal) in cells treated as described in (A). Bars indicate mean ± SE after analysis of 5 cells (>20 KTs were counted per cell). * *P*<0.01 versus DMSO treated cells. ** *P*<0.01 versus GL2 siRNA transfected cells.

In support of this view, and in agreement with recent independent studies [20], [21], we found that siRNA-mediated depletion or inhibition of Aurora B led to a significant reduction of the hMps1 signal at KTs (Figures 3A and B). This suggests that Aurora B positively regulates hMps1 localization to KTs, which may then promote hMps1 hyperphosphorylation and activation through an increase in the local concentration of hMps1.

### hMps1 autophosphorylation sites resemble the Plk1 consensus motif

**Figure 4:**
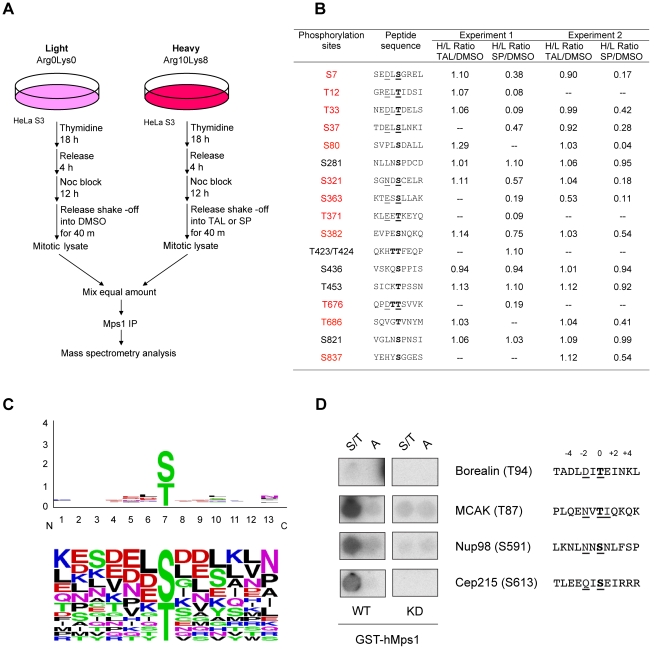
Potential Plk1 sites are hMps1 autophosphorylation sites. (A) Schematic representation of the SILAC experimental protocol used to test the effect of the small molecule inhibitors TAL and SP600125 on hMps1 phosphorylation *in vivo*. (B) Table showing the relative phosphorylation levels, as identified by mass spectrometry, after treatment with TAL or SP600125. Phosphorylation sites responsive to SP600125 treatment are shown in red, phosphoacceptor residues are shown in bold and residues matching the proposed hMps1 consensus motif are underlined. The table shows the results from two independent experiments. Hyphens indicate that the phosphopeptide was not identified. (C) Web logo analysis of hMps1 autophosphorylation sites (upper panel). Logos were created using Web logo 2.8.2 (http://weblogo.berkeley.edu/) [59]. In the lower panel the corresponding frequency plot is reported. (D) Previously identified phosphorylation sites matching the broadened Plk1 consensus but being unresponsive to Plk1 inhibition or depletion [36] were chosen for peptide spot kinase assays. From left to right: peptide spots (phosphoacceptor S/T to the left; alanine substitution A to the right), protein name, phosphorylated residue and target peptide. Phosphoacceptor residues are shown in bold, residues matching the broadened Plk1 consensus motif are underlined. Membranes were either incubated with wild-type (WT) or kinase-dead (KD) GST-hMps1. Borealin (T94) was included as control.

Taking into account the large number of apparent Plk1 phosphorylation sites identified on endogenous hMps1 (Fig. 1C), we were surprised to find that the Plk1 inhibitor TAL failed to detectably influence hMps1 hyperphosphorylation (Fig. 2C). To explore which, if any, of the putative Plk1 sites on hMps1 were sensitive to TAL treatment *in vivo*, we combined mass spectrometry with SILAC (stable isotope labeling with amino acids in cell culture) (Fig. 4A). In parallel experiments, we inhibited hMps1 itself using SP600125. HeLa S3 cells labeled with amino acids made of light or heavy isotopes were released from a Thymidine arrest into Nocodazole. Mitotic cells were then collected by shake-off and released for 40 minutes into fresh medium containing kinase inhibitors (TAL or SP600125) or DMSO for control. After mixing equivalent amounts of lysates from drug-treated and control cells, endogenous hMps1 was immunoprecipitated, resolved by SDS-PAGE, and in-gel digested by trypsin. Following TiO_2_ enrichment of phosphopeptides, phosphorylation sites were analyzed and quantified by mass spectrometry. Remarkably, phosphorylation at most potential Plk1 sites was not detectably reduced upon TAL treatment (Fig. 4B). Only phosphorylation at S363 was reduced in response to both TAL and SP600125, and phosphopeptides corresponding to T371 and T676 could not be identified in the TAL treated samples. These results strongly argue that Plk1 is not responsible for most of the phosphorylations on putative ‘Plk1 consensus’ sites on hMps1. In striking contrast, inhibition of hMps1 caused a marked reduction in phosphorylation on 12 sites (S7, T12, T33, S37, S80, S321, S363, T371, S382, T676, T686, S837) (Fig. 4B). SP600125 was originally developed as an inhibitor of JNK [45] and has recently been shown to also inhibit Aurora B *in vitro* [21]. Hence SP600125 cannot be considered a specific inhibitor of hMps1. However, we emphasize that phosphorylation at 4 putative Cdk1 sites was not affected by SP600125 treatment (Fig. 4B); furthermore, *in vitro* kinase assays revealed no influence of SP600125 on Plk1 kinase activity (data not shown). Considering the reported inhibitory effect of SP600125 on Aurora B, it is possible that SP600125 treatment leads to an indirect inactivation of hMps1 via a lack of Aurora B-dependent recruitment to kinetochores. However, we emphasize that similar results to those shown in Figure 4B were also obtained when using the more specific Mps1 inhibitor Mps1-IN-1 ([16], Table S2). Thus, the most straightforward interpretation of the above data is that SP600125 indeed inhibited hMps1. Collectively, our SILAC data strongly suggests that phosphorylation of several putative ‘Plk1 sites’ on hMps1 is not brought about by Plk1 but by hMps1 itself. Recently, Tyler et al. reported numerous hMps1 autophosphorylation sites [32], but several of these could not be confirmed here as *in vivo* hMps1 phosphorylation sites. On the other hand, 7 additional sites (S15, T288, T360, S362, T564, S682, S742) not identified in our SILAC analysis were also reported as *in vivo* autophosphorylation sites in recent publications [31], [32], [46]. A Web logo representation of all autophosphorylation sites identified *in vivo*, either here or previously (see Table S3), confirms that hMps1 phosphorylation sites demonstrate a propensity for E/D/N/Q at the −2 position (11 of 19 sites), very similar to the Plk1 consensus motif (Fig. 4C) [36].

The resemblance of the broadened Plk1 and proposed hMps1 consensus motifs prompted us to re-analyze a recently generated data set of the Plk1-dependent and spindle-associated phosphoproteome [36]. In particular, we searched this data set for phosphorylation sites that were unresponsive to chemical inhibition or knock-down of Plk1 in SILAC experiments but contained E/D/N/Q at the −2 position [36]. Using recombinant GST-hMps1 for phosphorylation of peptides spotted onto membranes, we could indeed show that several of these *in vivo* phosphorylation sites not only conform to the proposed hMps1 consensus motif but are also phosphorylated by hMps1 *in vitro*. These include MCAK (T87), Nup98 (S591) and Cep215 (S613) (Figure 4D).

## Discussion

In this study we examined *in vivo* hMps1 phosphorylation by mass spectrometry and investigated candidate upstream kinases for their possible involvement in hMps1 phosphorylation. Although a number of the hMps1 phosphorylation sites analyzed here were also identified independently by other groups [29], [30], [32], [33], [47], we emphasize that our study provides *in vivo* evidence for phosphorylation of several sites that had previously been identified only *in vitro*. Our biochemical analysis of hMps1 in mitotic cells indicates that Aurora B kinase and hMps1 itself are required for hMps1 hyper-phosphorylation during mitosis. Given that the mitotic upshift of hMps1 correlates with increased kinase activity [14], it is likely that hMps1 and/or Aurora B regulate the *in vivo* activity of hMps1. While hMps1 readily phosphorylated itself *in vitro*, only marginal *in vitro* phosphorylation of hMps1 by Aurora B was observed. Thus, it appears plausible that Aurora B causes mitotic hyperphosphorylation of hMps1 through an indirect mechanism. In line with evidence from *Xenopus* [48] and recent studies using the novel inhibitors Reversine and AZ3146 [20], [21], our immunofluorescence analysis indicated that the association of hMps1 with KTs was significantly reduced in response to an impairment of Aurora B function.

In addition to encompassing most of the previously identified phosphorylation sites (Table S1), our study reveals a number of novel sites. Interestingly, we found that 12 phosphorylation sites within hMps1 represent autophosphorylation sites. Weblogo analysis on these SP600125-sensitive sites and autophosphorylation sites reported by other groups showed that hMps1 phosphorylation sites have a preference for E/D/N/Q at the −2 position. This demonstrates that hMps1 and Plk1 are able to recognize similar substrate consensus motifs. In agreement with this conclusion, a recent survey of kinase specificities in *Saccharomyces cerevisiae* revealed a preference for D/E at the −2 position for yeast Mps1 [49]. Moreover, several published Mps1 substrates have a E/D/N/Q at −2 position, including Borealin (T94) [25], Ndc80 (S4, T38, T248, T252) [50], Dam1 (S221) [51], Spc29 (T18, T159, S187, T240) and Cdc31 (T110) [52], [53]. This list can further be extended by the substrates MCAK (T87), Nup98 (S591) and Cep215 (S613) presented in this study.

Although both hMps1 and Plk1 are important for mitotic progression and the fidelity of chromosome congression, the known roles of these kinases are widely different. While disruption of hMps1 function interferes with SAC activity and thus accelerates traverse through mitosis, inhibition of Plk1 causes a SAC-dependent mitotic arrest. Thus, it may appear surprising that at least some substrates of hMps1 and Plk1 share a similar consensus motif. *In vivo*, substrate specificity of any kinase is strongly influenced by subcellular localization. Moreover, the interactions between kinases and their substrates are influenced also by molecular features (in either kinase and/or substrate) that reside outside of the interaction domain between consensus motif and catalytic site. Nevertheless, our present data suggest that hMps1 and Plk1 share at least some common physiological substrate(s). Thus, the access of the two kinases to these substrates is expected to be tightly regulated in time and space. In future studies, it will be interesting to explore the possibility that hMps1 and Plk1 converge on a set of common physiological substrates during mitotic progression.

## Materials and Methods

### Cell culture and drug treatments

HeLa S3 cells were routinely maintained in DMEM (Invitrogen) supplemented with 10% FBS and penicillin-streptomycin (100 IU/ml and 100 mg/ml, respectively, GIBCO). Thymidine was used at 2 mM, Nocodazole at 100 ng/ml, SP600125 at 20 µM, Reversine at 0.5 µM, Mps1-IN-1 at 1.0 µM (kindly provided by Nathanael S. Gray), ZM447439 at 5 µM, TAL at 1 µM, Roscovitine at 100 µM, U0126 at 20 µM, and MG132 was used at 20 µM.

### Plasmids and recombinant protein production

For expressing recombinant hMps1, wild type and kinase dead hMps1 cDNA was amplified and cloned into the pVL1393 baculovirus transfer vector (Pharmingen) with an N-terminal GST tag, and then expressed in Sf9 cells following the manufacturer's instructions. Recombinant His-Plk1 [54] and MBP-BubR1 [43] have been described previously.

### Antibodies

Monoclonal anti-hMps1-N1 [14] and anti-BubR1 [43] antibodies have been previously described. Anti-α-tubulin (DM1A, Sigma) and ACA (Immunovision, Springdale, AR) were obtained commercially. For all Western blotting, signals were detected using HRP-conjugated anti-mouse or anti-rabbit antibodies (Pierce).

### Kinase assays

*In vitro* phosphorylation of recombinant kinase dead GST-hMps1 was carried out at 30°C using 300 ng of recombinant hMps1-KD and 100 ng of each kinase in 40 µl of kinase reaction buffer (50 mM Tris-HCl pH7.5, 10 mM MgCl_2_, 0.5 mM DTT, 10 µM ATP, 5 µCi γ-^32^P-ATP). Reactions were stopped after 30 min by addition of sample buffer. Samples were then resolved by SDS-PAGE and visualized by autoradiography. Recombinant active Cdk1/Cyclin B, MAPK (Erk2) and Aurora B kinase were purchased from Invitrogen.

### SILAC labeling with ^13^C_6_^15^N_4_-L-arginine and ^13^C_6_^15^N_2_-L-lysine

HeLa S3 cells were cultured in DMEM formulated with either unlabeled L-lysine and L-arginine or labeled ^13^C_6_^15^N_4_ -L-arginine and ^13^C_6_^15^N_2_-L-lysine (Cambridge Isotope Laboratories) at the concentration of 44 and 86 µg/ml respectively, and supplemented with 10% dialyzed fetal bovine serum, 50 units/ml penicillin, and 50 µg/ml streptomycin. Unlabeled and labeled HeLa cells were synchronized in mitosis using a sequential Thymidine block/release, Nocodazole block protocol. Unlabelled mitotic cells were collected and released into MG132 plus DMSO; in parallel, labeled mitotic cells were collected and released into MG132 plus TAL or SP600125. After 40 minutes, both sets of cells were collected and equal amount of cell lysates (∼15 mg each) were mixed together. Endogenous hMps1 was then immunoprecipitated using the anti-hMps1-N1 mAb. Samples were separated by a 4–12% NuPAGE gel (Invitrogen).

### Mass spectrometry and peptide identification

Coomassie Brilliant Blue (CBB)-stained protein bands were in-gel digested by trypsin (sequencing grade, Roche, Germany) essentially as described [55] and phosphorylated peptides were enriched using TiO_2_ affinity purification with glycolic acid as a modifier [56]. Subsequently, tryptic peptides were analyzed by nanoLC-MS/MS using a nanoACQUITY ultra performance liquid chromatography system (Waters, U.K.) coupled to an LTQ-Orbitrap (Thermo, Germany). Samples were injected onto a silica capillary column (New Objective, U.S.A.) packed with 3-µm ReproSil-Pur C18-AQ (Dr. Maisch GmbH, Germany). Peptides were separated by a stepwise 110-min gradient of 0–100% between buffer A (0.2% formic acid in water) and buffer B (0.2% formic acid in acetonitrile) at a flow rate of 200 nL/min. The mass spectrometer was operated in data dependent MS/MS mode to automatically switch between MS survey and MS/MS fragmentation scans of five most abundant precursor ions. Peak lists were generated using DTA supercharge [57] and searched using the Mascot (Matrix Science, UK) software package against the human International Protein Index (IPI) database (http://www.ebi.ac.uk/IPI/IPIhelp.html) with carbamidomethyl cysteine as a fixed modification and oxidized methionine, phosphorylation (S,T,Y), ^13^C_6_^15^N_4_ -L-arginine, and ^13^C_6_^15^N_2_-L-lysine as variable modifications. Searches were performed with a precursor mass tolerance of 5 ppm and fragment ion tolerances of 0.7 Da and identified phosphorylation sites were further validated by visual inspection of MS/MS spectra. For phosphopeptide quantification, the ratios between the monoisotopic peaks of labeled and unlabeled forms of phosphopeptides were calculated by MSQuant [57].

### Immunofluorescence microscopy, image processing and quantification

HeLa S3 cells grown on coverslips were fixed with a pre-extraction-fixation method using PHEM buffer [58]. Samples were examined on a Deltavision microscope (Applied Precision), with optical sections acquired 0.2 µm apart in the Z-axis. Deconvolved images from each focal plane were projected into a single picture using Softworx (Applied Precision). Images were taken at identical exposure times within each experiment, acquired as 24-bit RGB images, and processed in Adobe Photoshop. Images shown in the same panel have been identically scaled. Measurement of KT intensities was performed in ImageJ (http://rsb.info.nih.gov/ij/) on non-deconvolved images. Quantification of KT intensities was performed as previously described [43]. Essentially, a circular region with fixed diameter was centered on each KT, and unless indicated otherwise, anti-centromere antibody (ACA) intensity was measured in the same region and used for normalization (after subtraction of background intensity measured outside the cell).

### Peptide spotting assay

Peptide arrays were generated using standard F-moc chemistry on a MultiPep robotic spotter (Intavis) following the manufacturer's instructions. Peptides synthesized and immobilized on cellulose membranes were tested for phosphorylation by human GST-Mps1. Dried membranes were first washed in ethanol and then hydrated in kinase buffer (50 mM Tris-HCl pH7.5, 10 mM MgCl_2_, 100 mM NaCl, 1 mM DTT, 100 µM NaF, 1 mM Sodium Ortho-Vanadate) for 1 h, followed by overnight blocking in kinase buffer with 0.5 mg/ml BSA. The next day, the membrane was blocked again with kinase buffer containing 1 mg/ml BSA and 50 µM cold ATP at RT for 1 h. The blocking buffer was subsequently replaced with kinase reaction buffer containing 0.2 mg/ml BSA, 45 µCi/ml [γ-32P]-ATP and 50 µM cold ATP in the presence of recombinantly expressed GST-Mps1-WT or GST-Mps1-KD (kinase dead) for 3 h on a shaker at 30°C (5.0 ml reaction volume per membrane). Membranes were then washed extensively: 10×15 min in 1 M NaCl, 3×5 min in H_2_O, 3×15 min 5% H_3_PO_4_ , 3×5 min in H_2_O, and then sonicated overnight in 8 M urea, 1% SDS (w/v), and 0.5% (v/v) ß-mercaptoethanol to remove residual nonspecific radioactivity. The membranes were washed again with H_2_O, followed by ethanol and dried before being visualized by autoradiography.

## Supporting Information

Figure S1 ***In vitro*** **phosphorylation of recombinant GST-Mps1KD.** Substrate was incubated in either kinase buffer alone, or with Plk1, Cdk1, MAPK, Mps1 and Aurora B kinase. To show that kinases are active toward appropriate substrates, recombinant MBP-BubR1 was subjected to *in vitro* phosphorylation by Plk1 and Cdk1 kinase and His-MCAK to Aurora B kinase. The source of Mps1 kinase was immunoprecipitated Mps1 (Mps1 IP). The left panel shows CBB staining of the gel. The right panel shows the result of autoradiography.
(TIF)

Table S1 **Summary of identified hMps1 phosphorylation sites.** The first column summarizes phosphorylation sites, with novel *in vivo* sites identified in this study shown in bold. The second column shows short sequences adjacent to the phosphorylation sites (underlined residues). In the absence of definitive information on a particular phosphorylation site, potential alternative positions are shown. The third column shows the MASCOT score for each phosphopeptide identified in this study.
(PDF)

Table S2 **Relative phosphorylation levels after treatment with Mps1-IN-1.** Sites on hMps1 identified by mass spectrometry after treatment with Mps1-IN-1 are listed together with the corresponding peptide sequences and relative phosphorylation levels. The phosphoacceptor is shown in bold and residues matching the proposed hMps1 consensus motif are underlined.
(PDF)

Table S3 **Autophosphorylation sites for weblogo analysis.** Autophosphorylation sites identified in previous studies and in the present work were analyzed by Web logo software. 12 sites (S7, T12, T33, S37, S80, S321, S363, T371, S382, T676, T686, S837) were demonstrated as autophosphorylation by our SILAC analysis. As reported by other groups [1]–[3], 7 other sites (S15, T288, T360, S362, T564, S682, S742) not identified in our SILAC analysis are also *in vivo* autophosphorylation sites. Although S436 and S821 were also reported as autophosphorylation sites [1], our data demonstrate these two sites are not autophosphorylation sites. Residues highlighted in red belong to autophosphorylation sites matching the proposed hMps1 consensus motif.
(PDF)
