# Supplementary figures and images for: Quantitative Mass Spectrometry Analysis Reveals Similar Substrate Consensus Motif for Human Mps1 Kinase and Plk1
Source: PLoS One. 2011 Apr 13;6(4):e18793. doi: 10.1371/journal.pone.0018793 (PMC3076450; doi:10.1371/journal.pone.0018793)

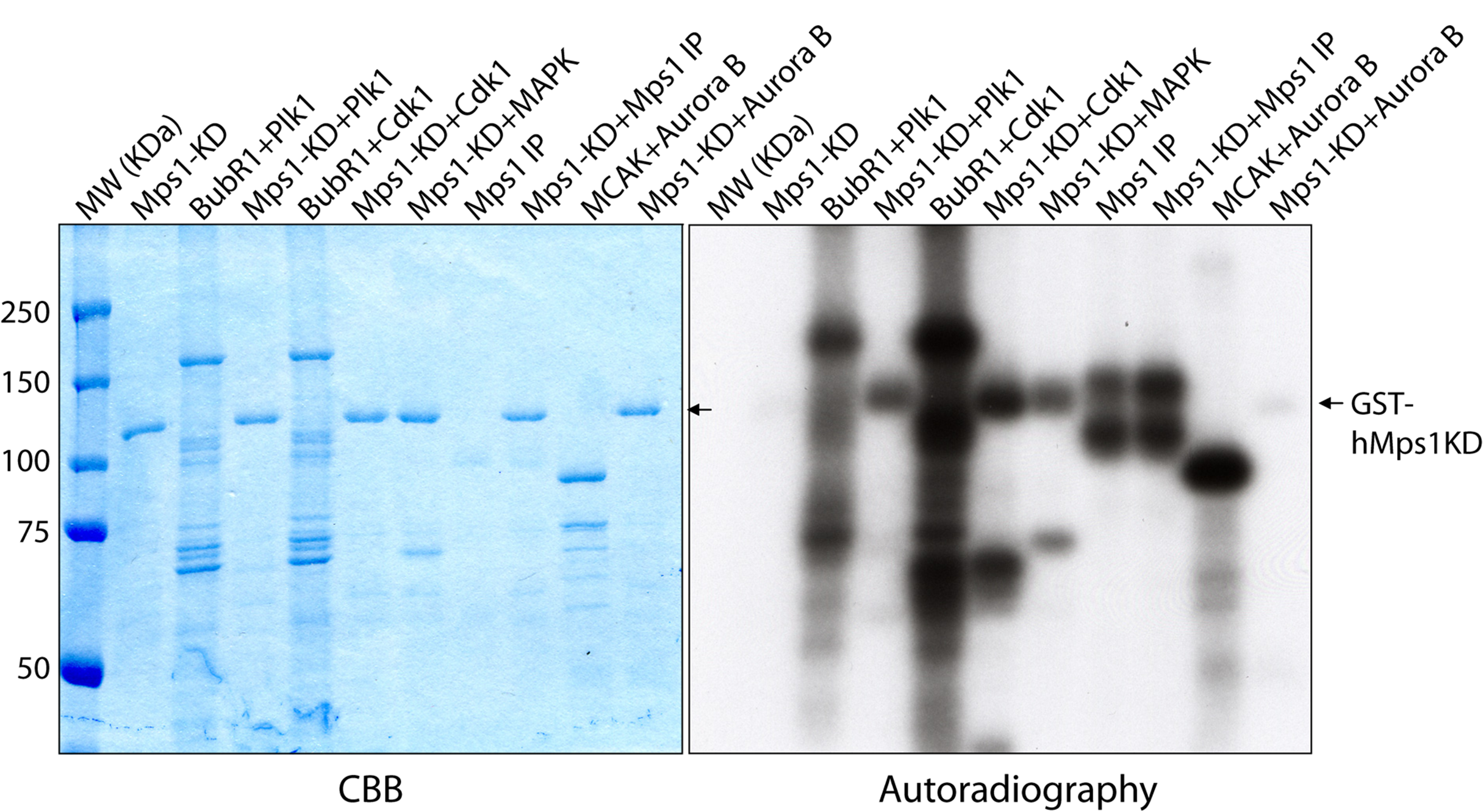

Supplement: Figure S1 — In vitro phosphorylation of recombinant GST-Mps1KD. Substrate was incubated in either kinase buffer alone, or with Plk1, Cdk1, MAPK, Mps1 and Aurora B kinase. To show that kinases are active toward appropriate substrates, recombinant MBP-BubR1 was subjected to in vitro phosphorylation by Plk1 and Cdk1 kinase and His-MCAK to Aurora B kinase. The source of Mps1 kinase was immunoprecipitated Mps1 (Mps1 IP). The left panel shows CBB staining of the gel. The right panel shows the result of autoradiography. (TIF) [file pone.0018793.s001.tif]
