# Supplementary material for: Quantitative Mass Spectrometry Analysis Reveals Similar Substrate Consensus Motif for Human Mps1 Kinase and Plk1
Source: PLoS One. 2011 Apr 13;6(4):e18793. doi: 10.1371/journal.pone.0018793 (PMC3076450; doi:10.1371/journal.pone.0018793)
Supplement: Table S1 — Summary of identified hMps1 phosphorylation sites. The first column summarizes phosphorylation sites, with novel in vivo sites identified in this study shown in bold. The second column shows short sequences adjacent to the phosphorylation sites (underlined residues). In the absence of definitive information on a particular phosphorylation site, potential alternative positions are shown. The third column shows the MASCOT score for each phosphopeptide identified in this study. (PDF) [file pone.0018793.s002.pdf]

Table S1. Summary of identified hMps1 phosphorylation sites.

Phosphorylation Site	Sequence	MASCOT Score	resembling Consensus	Reference
S7	DLSGR	39	Plk1	1, 6
T12	ELTID	29	Plk1	1, 9
S15	IDSIM	8	--	1, 9
T33	DLTDE	69	Plk1	1, 5, 6, 7, 9
S37	ELSLN	43	Plk1	1, 5, 6, 9
S49/T51	DNSGTVN		Plk1/--	6
S80	PLSDA	32	--	1, 5, 6, 9
S281	LNSPD	58	Cdk1	1, 4, 6, 7
T288	VKTDD	17	--	1, 9
S317	KPSGN		--	6
S321	NDSCE	68	Plk1	1, 5, 7
**S329**	LKSVQ	23	--	1
S345/346	EKSSEL		Plk1/Aur-B	6
T351	IITDS		--	6
S353	TDSIT		--	4
T360	NKTES	33	Plk1	1, 4, 5, 9
S362	TESSL	17	--	1, 9
S363	ESSLL	24	Plk1	1, 4, 5, 9
T371	EETKE	56	Plk1	1, 4
S382	PESNQ	50	--	1, 4
S393	RKSEC		Aur-B	5, 6, 7
**T418**	VNTQE	21	--	1
**T423/T424**	KHTTFE	9	Aur-B/--	1
S436	KQSPP	39	Cdk1	1, 3, 4, 5, 6
T453	CKTPS	77	Cdk1	1, 3
S455	TPSSN	28	--	1, 7
**T458**	SNTLD	68	--	1
Y462	DDYMS		--	5
T468	FRTPV		Cdk1	3, 4
T564	NQTLD	58	Plk1	1, 8
S582	QHSDK		--	8
T675	PDTTS		--	2, 9
T676	DTTSV	50	Plk1	1, 2, 4, 5, 8, 9
S677	TTSVV		--	2, 9
S682	KDSQV	50	Aur-B	1, 8
T686	VGTVN	55	--	1, 2, 5, 8, 9
S742	QISKL	46	Plk1	1, 8
T795	IQTHP		--	8
T805	KGTTE		Aur-B	8
T806	GTTEE		--	8
Y811	MKYVL		--	8
S821	LNSPN	46	Cdk1	1, 4, 5, 6, 7, 8
S824	PNSIL	8	--	1, 5, 7, 8
Y833/Y836/S837	TLYEHYSGG	55	--	1, 6
S845	NSSSS		Plk1	8
S847	SSSKT		--	8
T849	SKTFE		--	8

References:

1. this study

2. Mattison CP, Old WM, Steiner E, Huneycutt BJ, Resing KA, et al. (2007) Mps1 activation loop autophosphorylation enhances kinase activity. J Biol Chem 282: 30553-30561.

3. Kasbek, C., Yang, C.H., Yusof, A.M., Chapman, H.M., Winey, M. and Fisk, H.A. (2007). Preventing the degradation of mps1 at centrosomes is sufficient to cause centrosome reduplication in human cells. Mol Biol Cell 18, 4457-69.

4. Kang J, Chen Y, Zhao Y, Yu H (2007) Autophosphorylation-dependent activation of human Mps1 is required for the spindle checkpoint. Proc Natl Acad Sci U S A 104: 20232-20237.

5. Jelluma N, Brenkman AB, McLeod I, Yates JR, 3rd, Cleveland DW, et al. (2008) Chromosomal instability by inefficient Mps1 auto-activation due to a weakened mitotic checkpoint and lagging chromosomes. PLoS One 3: e2415.

6. Daub H, Olsen JV, Bairlein M, Gnad F, Oppermann FS, et al. (2008) Kinase-selective enrichment enables quantitative phosphoproteomics of the kinome across the cell cycle. Mol Cell 31: 438-448.

7. Dephoure, N., Zhou, C., Villen, J., Beausoleil, S.A., Bakalarski, C.E., Elledge, S.J. and Gygi, S.P. (2008). A quantitative atlas of mitotic phosphorylation. Proc Natl Acad Sci U S A 105, 10762-7.

8. Tyler RK, Chu ML, Johnson H, McKenzie EA, Gaskell SJ, et al. (2009) Phosphoregulation of human Mps1 kinase. Biochem J 417: 173-181.

9. Xu Q, Zhu S, Wang W, Zhang X, Old W, et al. (2009) Regulation of kinetochore recruitment of two essential mitotic spindle checkpoint proteins by Mps1 phosphorylation. Mol Biol Cell 20: 10-20.
