# Supplementary material for: Quantitative Mass Spectrometry Analysis Reveals Similar Substrate Consensus Motif for Human Mps1 Kinase and Plk1
Source: PLoS One. 2011 Apr 13;6(4):e18793. doi: 10.1371/journal.pone.0018793 (PMC3076450; doi:10.1371/journal.pone.0018793)
Supplement: Table S2 — Relative phosphorylation levels after treatment with Mps1-IN-1. Sites on hMps1 identified by mass spectrometry after treatment with Mps1-IN-1 are listed together with the corresponding peptide sequences and relative phosphorylation levels. The phosphoacceptor is shown in bold and residues matching the proposed hMps1 consensus motif are underlined. (PDF) [file pone.0018793.s003.pdf]

Table S2. Relative phosphorylation levels after treatment with Mps1-IN-1.

Phosphorylation Site	Peptide sequence	H/L ratio IN-1/DMSO
S7	MESEDL**S**GR	0.11
S37	FKNEDLTDEL**S**LNK	0.11
S281	VPVNLLN**S**PDCDVK	0.97
S321	DLVVPGSKPSGND**S**CELR	0.22
S436	Q**S**PPISTSK	1.25
