# Supplementary material for: Quantitative Mass Spectrometry Analysis Reveals Similar Substrate Consensus Motif for Human Mps1 Kinase and Plk1
Source: PLoS One. 2011 Apr 13;6(4):e18793. doi: 10.1371/journal.pone.0018793 (PMC3076450; doi:10.1371/journal.pone.0018793)
Supplement: Table S3 — Autophosphorylation sites for weblogo analysis. Autophosphorylation sites identified in previous studies and in the present work were analyzed by Web logo software. 12 sites (S7, T12, T33, S37, S80, S321, S363, T371, S382, T676, T686, S837) were demonstrated as autophosphorylation by our SILAC analysis. As reported by other groups [1]–[3], 7 other sites (S15, T288, T360, S362, T564, S682, S742) not identified in our SILAC analysis are also in vivo autophosphorylation sites. Although S436 and S821 were also reported as autophosphorylation sites [1], our data demonstrate these two sites are not autophosphorylation sites. Residues highlighted in red belong to autophosphorylation sites matching the proposed hMps1 consensus motif. (PDF) [file pone.0018793.s004.pdf]

Table S3. Autophosphorylation sites for weblogo analysis.

Phosphorylation Site	Sequence	Sequence window
**S7**	DLSGR	MESEDLSGRELTI
**T12**	ELTID	LSGRELTIDSIMN
**S15**	IDSIM	RELTIDSIMNKVR
**T33**	DLTDE	FKNEDLTDELSLN
**S37**	ELSLN	DLTDELSLNKISA
**S80**	PLSDA	KNSVPLSDALLNK
**T288**	VKTDD	PDCDVKTDDSVVP
**S321**	NDSCE	KPSGNDSCELRNL
**T360**	NKTES	ITLKNKTESSLLA
**S362**	TESSL	LKNKTESSLLAKL
**S363**	ESSLL	KNKTESSLLAKLE
**T371**	EETKE	LAKLEETKEYQEP
**S382**	PESNQ	EPEVPESNQKQWQ
**T564**	NQTLD	EEADNQTLDSYRN
**T676**	DTTSV	QMQPDTTSVVKDS
**S682**	KDSQV	TSVVKDSQVGTVN
**T686**	VGTVN	KDSQVGTVNYMPP
**S742**	QISKL	QIINQISKLHAII
**S837**	HYSGG	TLYEHYSGGESHN

References:

1. Jelluma, N., Brenkman, A.B., McLeod, I., Yates, J.R., 3rd, Cleveland, D.W., Medema, R.H. and Kops, G.J. (2008). Chromosomal instability by inefficient Mps1 auto-activation due to a weakened mitotic checkpoint and lagging chromosomes. PLoS One 3, e2415.

2. Tyler, R.K., Chu, M.L., Johnson, H., McKenzie, E.A., Gaskell, S.J. and Eyers, P.A. (2009). Phosphoregulation of human Mps1 kinase. Biochem J 417, 173-81.

3. Xu, Q., Zhu, S., Wang, W., Zhang, X., Old, W., Ahn, N. and Liu, X. (2009). Regulation of kinetochore recruitment of two essential mitotic spindle checkpoint proteins by Mps1 phosphorylation. Mol Biol Cell 20, 10-20.
